# Geneva calling: WHO resolution on mental disorders

**DOI:** 10.1186/1752-4458-6-6

**Published:** 2012-06-14

**Authors:** Corrado Barbui, Benedetto Saraceno

**Affiliations:** 1 WHO Collaborating Centre for Research and Training in Mental Health and Service Evaluation, Department of Public Health and Community Medicine, Section of Psychiatry, University of Verona, Verona, Italy; 2 Gulbenkian Global Mental Health Platform, Lisbon, Portugal, and WHO Collaborating Centre for Research and Training in Mental Health, Department of Psychiatry, University of Geneva, Geneva, Switzerland

## Abstract

**Background:**

A new resolution on mental, neurological and substance use disorders was adopted in January 2012 by the World Health Organization (WHO) Executive Board. The resolution urges WHO and Member States to collaborate in the development of a comprehensive mental health action plan, to be submitted for discussion and approval to the WHO World Health Assembly. This commentary aims at rising awareness on the risk that this resolution may not fulfil its potential.

**Discussion:**

Lack of political awareness and visibility of the resolution is a first major issue. Theoretically, Member States should be aware of the resolution and support its implementation at their respective national level, but in practice political commitment may not be high enough, and technical and financial resources made available may be limited. A second challenge is that the resolution suggests to work with Member States and technical agencies to promote academic exchange through which to contribute to policy-making in mental health. It is not straightforward, however, how such a statement may be effectively translated into action. A third key methodological aspect is how scientific evidence and factors other than scientific evidence will be handled. This seems particularly relevant in the field of mental health, where value-based decisions together with resource and feasibility considerations may be unavoidable.

**Summary:**

We argue that WHO and Member States should work together to increase the visibility of the resolution, ensuring that Ministries of Health and other relevant components of the health systems are aware of the resolution and its implications. As the resolution urges for academic exchange, WHO should develop a plan for an explicit, inclusive and open call for support and collaboration, so that partners willing to contribute are not kept out from the process. The production of an action plan for mental disorders should be based on scientifically sound methodology. Such a methodology should be transparently described, for example in a WHO process document, to make it clear how individual-level recommendations and policy-level guidance are developed. WHO should establish and maintain an open forum of experts, scientists, health officials and user groups worldwide to interact and agree on values, preferences, feasibility, acceptability, implementability, equity and economic issues that should inform the action plan.

## Background

**Figure 1:**
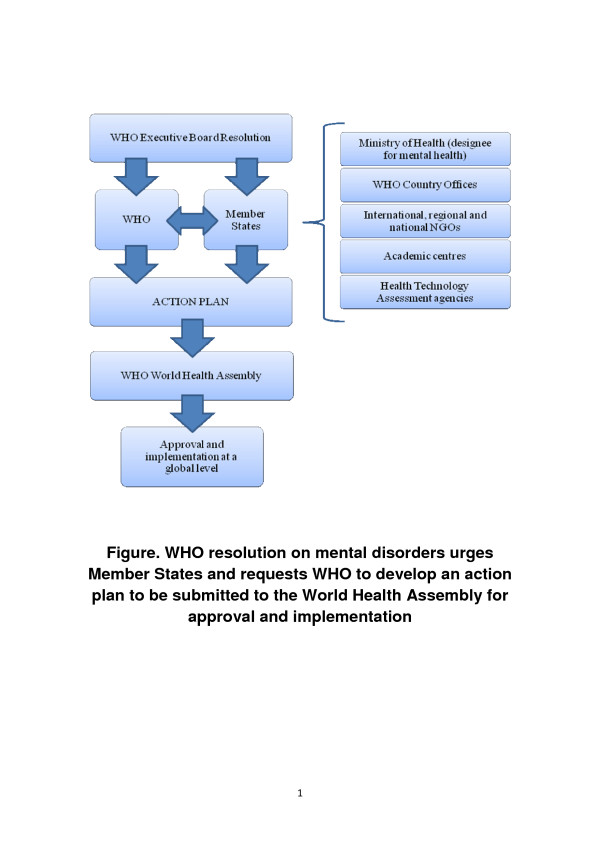
WHO resolution on mental disorders urges Member States and requests WHO to develop an action plan to be submitted to the World Health Assembly for approval and implementation.

At the recent 130th Executive Board (EB) session of the World Health Organization (WHO), a new resolution on mental, neurological and substance use disorders was adopted by Member States [1]. The resolution urges WHO and Member States to collaborate in the development of a comprehensive mental health action plan, to be submitted for discussion and approval to the WHO World Health Assembly (WHA) (see Table 1 for a definition of roles and responsibilities of WHO, WHA, EB and resolutions). Once approved by the WHA, the action plan should be implemented at global and regional levels (Figure 1). According to the resolution, involvement of Member States in the development of the action plan implies collaborations between WHO and Ministries of Health, or their designees, and also with non-governmental organizations (NGOs), academic centres and health technology agencies.

**Table 1:** Description of roles and responsibilities of the World Health organization (WHO), World Health Assembly, Executive Board and WHO resolutions

**WHO entity**	**Role and responsibility**
**World Health Organization (WHO) (www.who.int/)**	WHO is the directing and coordinating authority for health within the United Nations system. WHO’s Constitution came into force on 7 April 1948. The Organization is responsible for providing leadership on global health matters, shaping the health research agenda, setting norms and standards, articulating evidence-based policy options, providing technical support to countries and monitoring and assessing health trends.
**World Health Assembly (WHA) (www.who.int/ mediacentre/events/governance/wha/en/index.html)**	The World Health Assembly is the supreme decision-making body for WHO. It consists of every Minister of Health, or their designees, from 194 Member States. Its main function is to determine the policies of the Organization. Members of the WHA meet annually to discuss health topics, to set the WHO’s priorities, and to give suggestions for Member States. It similarly considers reports of the Executive Board, which it instructs in regard to matters upon which further action, study, investigation or report may be required.
**Executive Board (EB) (www.who.int/governance/ eb/en/index.html)**	The Executive Board is composed of 34 members technically qualified in the field of health. The main functions of the Board are to give effect to the decisions and policies of the WHA, to advise it and to facilitate its work.
**WHO Resolutions (http://apps.who.int/gb/or/)**	Resolutions are documents adopted by the WHA and by the EB after preliminary discussion, debate, and negotiations. The EB may recommend to the WHA the adoption of resolutions. Resolutions are not binding on Member States. Resolutions may urge Member States to take certain activities related to a certain health problem. Resolutions may also request WHO to perform certain activities. WHA resolutions are often very influential, affecting actions by governments and funding decisions by donors.

Indubitably, this resolution represents a great opportunity to increase the attention and resources given to mental healthcare globally, and to develop new innovative policies aimed at promoting mental health, preventing mental disorders and providing adequate treatment to those in need [2]. The opportunity seems particularly relevant and concrete on this occasion, as the resolution suggests an inclusive approach, with WHO, Member States, and a diverse spectrum of partners all urged to work together towards the common goal of developing a high-impact action plan.

However, a risk exists that this resolution, and the action plan that will be formulated accordingly, may not fulfil this potential. A risk exists that WHO and Member States will eventually produce a formal and politically-correct compilation of statements with a low potential to have an impact on mental health policies, service organization and care delivery worldwide. In this commentary we concisely report the reasons for such a risk, together with some suggestions for dealing with it.

## Challenges in implementing the WHO resolution on mental disorders

### Political awareness and international visibility

The resolution was issued in January 2012, and so far it has received little consideration worldwide. Theoretically, Member States should be aware of the resolution and support its implementation at their respective national level, and this support should reach the level of those who make decisions on policies and plans for mental disorders in individual countries. In practice, however, political commitment may not be high enough, technical and financial resources made available may be limited, and consequently the expectation that Ministries of Health will be automatically proactive is not that realistic, despite their formal approval of the resolution. Similarly, international and national organizations, including NGOs and donors, with a genuine interest in fostering mental healthcare programs in their own countries or globally, may simply be not aware of the resolution. This risk may not apply to international bilateral and multilateral donors or to international NGOs, which are in formal relation with WHO, but may be particularly high for national and local NGOs active in donor countries and in developing countries, which do not have habitual relations with WHO [3]. Lack of political awareness and visibility of the resolution, and of its potential, is a first major issue.

### Support and collaboration from member states and technical agencies

The resolution urges Member States to collaborate with WHO in the development of an action plan, and requests WHO to formulate such a plan, in collaboration with Member States. A critical aspect, related to the lack of awareness of the resolution, is how WHO may effectively interact with all the partners that may contribute. The resolution suggests to work with Member States and technical agencies to promote academic exchange through which to contribute to policy-making in mental health. It is not straightforward, however, how such a statement may be effectively translated into action. We argue that this aspect is not trivial, as it may dramatically affect the subsequent implementability of the whole action plan. Current lack of political awareness and visibility of the resolution, together with lack of a broad and highly visible call for collaboration at a global level, might represent major limitations for the whole process. Time is also an issue, as the resolution should be adopted by the WHA in May 2012, and the action plan is expected to be ready in 2013 [4].

### Scientific evidence, value-based decisions and feasibility considerations

A third important aspect that WHO and Member States may want to consider is how scientific evidence and factors other than scientific evidence will be handled. According to the resolution, the action plan should cover services, policies, legislation, plans, strategies and programs to promote mental health, prevent mental disorders, provide treatment, facilitate recovery, and empower persons with mental disorders to live a full and productive life in the community. This implies the production of a very diverse range of recommendations, including clinical practice guidelines (individual level) and health systems guidance (policy level). At both these levels scientific evidence is considered an extremely valuable tool, but it cannot be the only consideration [5,6]. This seems particularly relevant in the field of mental health, where value-based decisions together with resource and feasibility considerations may be unavoidable [7]. Therefore, according to how scientific evidence and other aspects are amalgamated together, it may be possible to formulate evidence-based or evidence-informed recommendations or policies, the difference being in the relative weight that is given to evidence and to factors other than the evidence. For example, it has recently been suggested that individual-level recommendations should be evidence-based, but policy-level guidance should be evidence-informed. The rationale for this would be that the contribution of aspects not related to the background evidence, including acceptability of policy options by stakeholders, implementation feasibility and equity, may be substantial [6]. Economic consequences may also play a key role: in Europe, one of the less visible consequences of the financial crisis has been for much increased involvement of the European Union in running national health systems with close supervision of national budgets [8]. Lack of a priori definition of these methodological aspects is expected to decrease the potential impact of the action plan in terms of acceptability by Member States and, if accepted and approved, in terms of implementability.

### The quality of being measurable

The resolution requests the development of an action plan with measurable outcomes. Although this seems a very sensible request, as WHO and Member States should definitely plan evaluation and monitoring activities with the aim of measuring the impact of the action plan in terms of pre-defined and agreed outcome measures, it nevertheless induces WHO and Member States to base recommendations not only on scientific evidence and values, preferences and feasibility issues, but also on their measurability, according to the philosophy of “what gets measured gets done”. We note that the quality of being measurable is certainly valuable for evaluation purposes, but may lead to the systematic exclusion of vital clinical practice recommendations and policy guidance. For example, ensuring that communication is clear, empathic, and sensitive to age, gender, culture and language differences, being friendly, respectful and non-judgmental at all times, using simple and clear language, are all simple recommendations that lack the quality of being measurable, but are considered essential components of any interaction with an individual suffering from mental disorders. At a policy level, the concept of equity may be difficult to translate into measurable outcomes [9], as well as the notion of respect of human rights of psychiatric service users [10,11].

### Harmonization with the UN convention on the rights of persons with disabilities

A fifth challenging aspect is how the action plan will be able to respect and reinforce the implications of the UN Convention on the Rights of Persons with Disabilities, an international human rights instrument of the United Nations intended to protect the rights and dignity of persons with disabilities [12]. The UN Convention came into force in 2008 and, as of February 2012, has 153 signatories and 110 parties, including the European Union which ‘concluded’ the treaty on 23 December 2010. Several aspects covered by the Convention may be extremely relevant in improving the lives of people with mental disabilities. For example, it promotes full inclusion and participation in community life and access to quality health care services as close as possible to people’s own communities. Considering that all over the world mental health services, if available, are often provided through mental health institutions which are associated with numerous human rights violations and poor health outcomes [13], the Convention may have huge implications in terms of deinstitutionalisation and the development of community based mental health and social services. Harmonization of the action plan with the UN Convention is therefore a challenging issue, as well as harmonization with other similar initiatives that may have a high impact in improving access to quality health care services worldwide [14].

## Improving the implementability of the WHO resolution on mental disorders

In order to increase the likelihood that this important resolution receives all the attention and consideration that it deserves, and that an action plan backed by solid methodology is timely developed and approved in 2013, and effectively implemented thereafter (Figure 1), WHO and Member States should consider following some simple steps.

First, they should work together to increase the visibility of the resolution, ensuring that Ministries of Health and other relevant components of the health systems are aware of the resolution and its implications. Most low- and middle-income countries have WHO Country Offices, which by definition have a close collaboration with the Ministries of Health. They should become the key champions of the resolution through its dissemination and promotion, and should offer support, collaboration and technical expertise towards its implementation. NGOs, academic centres, health technology agencies and other international and national agencies should similarly be given the possibility to contribute. User groups should be part of this consultation process, as they represent a key voice in service planning and decision-making processes.

Second, international and national scientific and professional organizations may also play a role. The resolution urges for academic exchange. WHO may choose to follow an implicit unstructured way to promote such exchange, but it is nevertheless possible to develop a plan for an explicit, inclusive and open call for support and collaboration, so that partners willing to contribute are not kept out from the process. A web-based resource can be easily implemented to give information on the resolution and offer the possibility to potential partners to upload information on how they may contribute. WHO may this way create a network of agencies that would work, under its leadership, to implement the recommendations of the resolution, with an active and collaborative participation in the development of the action plan. By fostering the integration of the activities of all these agencies, offering coordination with institutional representatives of Ministries of Health, WHO would reinforce its role and leadership in making more coherent, and conflict-of-interest free, the fragmented world of global mental health [3].

Third, the production of an action plan for mental disorders should be based on scientifically sound methodology. Such a methodology should be transparently described, for example in a WHO process document, to make it clear how individual-level recommendations and policy-level guidance are developed. In the area of mental disorders WHO has recently produced evidence-based recommendations using the GRADE methodology [7,15]. The same methodology may be applied to the action plan, or the action plan may refer to the work that WHO has already done within its mhGAP programme [15]. For the production of policy-level recommendations, which are expected to constitute the most relevant part of the action plan, a similar framework should urgently be adopted. For example, WHO has recently commissioned the production of a handbook outlining approaches to develop health systems guidance [16-18], and these approaches may be used and adapted by WHO and Member States for the production of policy-level recommendations within the action plan. We note that setting a high methodological standard would give added value to the action plan not only scientifically, but also in terms of acceptability and implementability. It is expected that many recommendations will consider aspects not related to the evidence base, thus requiring an even more explicit approach based on dialogue and consensus. WHO should take a leading role in this process by coordinating an open forum of experts, scientists and health officials worldwide to interact and agree on values, preferences, feasibility, acceptability, implementability, equity and economic issues that should inform the action plan [3]. WHO should make the results of such exchanges available for consultation on a web-based resource.

Fourth, it should be noted that to set up an effective and broad consultation process, based on a systematic process for evidence synthesis and stakeholder engagement, the WHO secretariat would need resources and time. It would be essential that Member States make immediately available financial resources to WHO for this purpose. The time (one year) given by the resolution to WHO secretariat to set up the plan is probably too short to follow all the processes that we are envisaging and recommending in this paper: would it not have been better for Member States and WHO to request the plan by the year 2014?

## Competing interests

Corrado Barbui acted as WHO consultant during the development of the mhGAP Action Program and currently provides methodological support to the WHO Department of Mental Health and Substance Abuse, Geneva, Switzerland, in the development of evidence-based recommendations using the GRADE approach.

Benedetto Saraceno used to be Director of the WHO Department of Mental Health and Substance Abuse, Geneva, Switzerland, for more than 10 years.

## Authors’ contributions

CB drafted the first version of the manuscript. BS commented and refined the manuscript in preparation for submission. CB and BS approved the final version to be published. Both authors read and approved the final manuscript.
